# The role of miR-26a and miR-30b in HER2+ breast cancer trastuzumab resistance and regulation of the *CCNE2* gene

**DOI:** 10.1038/srep41309

**Published:** 2017-01-25

**Authors:** Eduardo Tormo, Anna Adam-Artigues, Sandra Ballester, Begoña Pineda, Sandra Zazo, Paula González-Alonso, Joan Albanell, Ana Rovira, Federico Rojo, Ana Lluch, Pilar Eroles

**Affiliations:** 1 INCLIVA Biomedical Research Institute, 46010 Valencia, Spain; 2 Pathology Department, IIS-Fundación Jiménez Díaz, 28040 Madrid, Spain; 3 Cancer Research Program, IMIM (Hospital del Mar Research Institute), 08003 Barcelona, Spain; 4 Medical Oncology Department, Hospital del Mar, 08003 Barcelona, Spain; 5 Pompeu Fabra University, 08002 Barcelona, Spain; 6 Oncology and Hematology Department, Hospital Clínico Universitario, 46010 Valencia, Spain

## Abstract

A subset of HER2+ breast cancer patients manifest clinical resistance to trastuzumab. Recently, miR-26a and miR-30b have been identified as trastuzumab response regulators, and their target gene *CCNE2* seems to play an important role in resistance to trastuzumab therapy. Cell viability was evaluated in trastuzumab treated HER2+ BT474 wt (sensitive), BT474r (acquired resistance), HCC1954 (innate resistance), and MDA-MB-231 (HER2−) cell lines, and the expression of miR-26a, miR-30b, and their target genes was measured. BT474 wt cell viability decreased by 60% and miR-26a and miR-30b were significantly overexpressed (~3-fold, *p* = 0.003 and *p* = 0.002, respectively) after trastuzumab treatment, but no differences were observed in resistant and control cell lines. Overexpression of miR-30b sensitized BT474r cells to trastuzumab (*p* = 0.01) and *CCNE2*, was significantly overexpressed after trastuzumab treatment in BT474r cells (*p* = 0.032), but no significant changes were observed in sensitive cell line. When *CCNE2* was silenced BT474r cell sensitivity to trastuzumab increased (*p* = 0.03). Thus, the molecular mechanism of trastuzumab action in BT474 cell line may be regulated by miR-26a and miR-30b and *CCNE2* overexpression might play an important role in acquired trastuzumab resistance in HER2+ breast cancer given that resistance was diminished when *CCNE2* was silenced.

Breast cancer is a complex and heterogeneous disease that accounts for 25% of all cancer cases and 15% of all female deaths from cancer worldwide^1^. Approximately 20–30% of breast tumors overexpress HER2^2^ and this subtype is characterized by high rates of cell proliferation and metastasis, a poor prognosis, low overall survival, and variable chemotherapy response^3^. However, the HER2 receptor is a target for personalized and directed therapy with monoclonal antibodies such as trastuzumab (Herceptin). This therapy blocks the function of the HER2 receptor (and related pathways) which promote migration, angiogenesis, and cell growth and survival, and thus prolongs patient survival and decreases the rate of tumor development^4^. Despite this, about 40% of metastatic patients present innate trastuzumab-resistance, and most patients develop acquired resistance within the first year of treatment^4^,^5^. Therefore, better knowledge of the molecular mechanisms of trastuzumab action is crucial for improving HER2+ breast cancer treatment in an attempt to overcome these resistance problems.

It has been previously described that trastuzumab blocks HER2 signaling by preventing HER2 homo/heterodimerization, thus inducing antibody-dependent cell-mediated cytotoxicity, promoting HER2 receptor internalization, and inhibiting its cleavage, which in turn blocks the MAPK, mTOR, and PI3K/Akt pathways^6^. However, there are no validated markers for resistance to HER2-targeted trastuzumab treatment. Little is known about the role of miRNAs in trastuzumab response, although some studies have identified miRNAs involved in carcinogenesis, malignancy, prognosis, and treatment response in different cancers^7^. For example, miR-34a and miR-221/222 are involved in the response to docetaxel and tamoxifen in breast cancer^8^, and miR-10b and miR-21 overexpression are associated with poor outcomes in breast cancer. Ichikawa and colleagues demonstrated that miR-26a and miR-30b are implicated in trastuzumab response by identifying them as trastuzumab-inducible miRNAs that are overexpressed in HER2 positive cell lines treated with trastuzumab^9^. However, the exact role of this overexpression in sensitivity or resistance to trastuzumab is still unexplained.

Following on from this work, our group focused on how miR-26a and miR-30b are implicated in this response to trastuzumab by examining the differences in miRNA expression in trastuzumab-sensitive, acquired-resistance, or innate-resistance HER2+ breast cancer cell lines. In addition, we also screened genes which may be targets of these miRNAs and which could also be involved in treatment response. We checked the regulation of *CCNE2* (Cyclin E2) by these miRNAs and its expression in different HER2+ breast cancer cell lines because this gene has been previously implicated in acquired trastuzumab resistance^10^ and is a possible miR-30b target^9^. We also describe possible miR-30b and miR-26a target genes related to the cell cycle and apoptosis that are likely implicated in trastuzumab response, and thus go on to suggest potential biomarkers which may be able to identify trastuzumab resistance in early treatment phases.

## Materials and Methods

### Cell lines and treatment

The HER2-positive breast cancer cell lines BT474 wt, BT474r, and HCC1954, were obtained from F.R.’s group at the *Fundación Jiménez Díaz* Hospital (Madrid) and the HER2-negative MDA-MB-231 breast cancer cell line was obtained from J.A.’s group at the IMIM, Hospital del Mar Research Institute (Barcelona). All the cell lines were grown at 37 °C with 5% CO_2._ The MDA-MB-231, BT474 wt, and BT474r cell lines with acquired resistance, generated by culture with 15 μg/ml trastuzumab for 6–12 months^11^, were grown in Dulbecco Modified Eagle medium nutrient mixture F-12 (DMEM/F12) with 2.5 mM L-Glutamine and 15 mM HEPES (Gibco), supplemented with 10% FBS and 1% penicillin-streptomycin. HCC1954 was cultured in RPMI 1640 medium (Gibco) containing 10% FBS, 1% penicillin-streptomycin, and 1% L-glutamine. Trastuzumab (Herceptin^®^, Roche) was used at 15 μg/ml.

### RNA isolation

Total RNA containing small RNAs was isolated using mirVana miRNA isolation kit (Ambion, Austin, Texas) according to the manufacturer’s protocol. The concentration and purity of the RNA obtained was measured as the OD260/280 ratio using a GeneQuant pro spectrophotometer (GE, Healthcare).

### Transfection

Cell lines were transfected either with 50 nM hsa-miR-26a-5p or hsa-miR-30b-5p mirVana mimic or inhibitor miRNAs (Ambion), and, where relevant, with 50 nM or 100 nM *CCNE2* siRNA (#AM16708, Thermofisher), using the TransIT-X2 Dynamic Delivery System reagent (Mirus), following the manufacturer’s instructions.

### Cell viability

BT474 wt, BT474r, HCC1954, and MDA-MD-231 cells were cultured in 96-well plates and treated with 15 μg/ml trastuzumab for 7 days with or without prior transfection with miRNA mimics/inhibitors or siRNA. Cell viability was measured using a MTT-based Cell Growth Determination Kit (#GDC1; Sigma). MTT was added to each well under sterile conditions (to a final concentration of 10% of the total volume) and the plates were incubated for 4 h at 37 °C. Formazan crystals were dissolved in the solubilization solution at a 1:1 ratio and, starting from yellow MTT, formed purple crystals via succinate dehydrogenase only in viable cells. Absorbance was measured at 570 nm in a microplate reader with background correction at 690 nm. The significance of any differences were assessed using the Student t test.

Sensitivity or resistance to trastuzumab was assessed using the following equations: Log (N_t_) = (Log2/DT) T + Log (N_0_) and fold change in growth rate = DT with drug/DT without drug, where DT = doubling time, N_t_ = number of cells after treatment, T = time of treatment, and N_0_ = number of cells before treatment. Cell lines with more than a 20% decrease in cell number in response to trastuzumab treatment were considered to be trastuzumab-sensitive^12^.

### Flow cytometry

BT474 wt and BT474r cell lines were cultured in 6-well plates and treated with 15 μg/ml trastuzumab for 7 days with or without prior transfection with hsa-miR-30b-5p mirVana miRNA mimic. The cells were labelled with 0.5 mg/ml annexin-V conjugated to the FITC fluorochrome and 0.5 mg/ml of propidium iodide (PI) using the FITC Annexin V Apoptosis Detection Kit I (BD Pharmingen™). The cells were incubated for 15 minutes in the dark at room temperature, followed by the addition of 400 μl of binding buffer. Annexin V binds apoptotic cells because of phosphatidylserine membrane residue translocation, and PI is incorporated because of loss of plasma membrane integrity. The amount of PI and annexin V labelling was analyzed by flow cytometry using a FACSVerse™ flow cytometer (BD) to determine cell viability by measuring the percentage of apoptotic cells present.

For cell cycle analysis, aliquots of 2 × 10^6^ cells were kept on ice prior to fixation. The cells were washed twice with cold PBS, decanted, and the pellet was resuspended before the addition of ethanol. The cells were then fixed by slowly pipetting 1 ml of 80% ice-cold ethanol into the cell suspension while vortexing; the cells were left in ethanol at −20 °C for 2 hours and were then washed twice with cold PBS. Finally they were resuspended in 1 ml freshly-made DAPI/TX-100 solution and incubated for 30 minutes at room temperature before analysis by flow cytometry (excitation at 405 nm and emission at 450 nm).

### Gene and miRNA expression by qRT-PCR

The expression of *CCNE2* (cyclin E2), *CASP3* (caspase 3), and *APAF1* (apoptotic peptidase activating factor 1) mRNA and levels of miR-26a and miR-30b were determined by real-time qPCR. First, 1000 ng RNA was retro-transcribed to cDNA using a High-Capacity cDNA Reverse Transcription kit (Applied Biosystems) and a TaqMan^®^ MiRNA Reverse Transcription kit; cDNA was then synthesized at 25 °C for 10 min and 37 °C for 2 hours for mRNA retro-transcription and 16 °C for 30 min, 42 °C for 30 min, and 85 °C for 5 min for miRNA retro-transcription, and was then stored at −20 °C until use. TaqMan^®^ primers for housekeeping miRNA *RNU43* as well as miR-26a and miR-30b were obtained from Applied Biosystems. Transcript levels were detected using a 9700HT Fast Real-Time PCR system (Applied Biosystems) and PCR reactions were performed with a TaqMan^®^ Universal Master Mix (Applied Biosystems) and TaqMan^®^ 20× assay following the manufacturer’s protocol. PCR conditions were: 50 °C for 2 min, 95 °C for 10 min, 40 cycles of 95 °C for 15 sec, and 60 °C for 1 min for gene expression, or 95 °C for 10 min, followed by 40 cycles of 95 °C for 15 sec, and 60 °C for 1 min for miRNA expression.

Results were normalized according to the expression of housekeeping *GAPDH* mRNA or *RNU43* miRNA. The threshold cycle value (C_T_) was determined for each measurement and mRNA or miRNA expression was calculated relative to the control using the comparative critical threshold (2−ΔΔC_T_) method where: ΔC_T_ = target C_T_ minus the housekeeping control C_T_, ΔΔC_T_ = sample ΔC_T_ minus the baseline (non-treated cells) ΔC_T_, and the relative expression level = 2^ΔΔCT^. Each experiment was performed in technical and biological triplicate.

### Western immunoblotting

Cells were seeded and then exposed to the indicated concentration of trastuzumab. After treatment and/or transfection experiments, cells were washed twice with cold PBS, and the monolayer was scraped into 1 ml of Pierce RIPA buffer (Thermo #89900). The lysates were transferred to a clean microfuge tube, placed on ice for 15 min, and centrifuged for 10 min at 14,000 rpm. The supernatant was transferred to a clean microfuge tube, and the protein concentration was determined. Protein extracts (40 μg) were boiled in Laemmli buffer and resolved on a 10% SDS-polyacrylamide gel, before transfer onto a nitrocellulose membrane. Membranes were blocked in 5% BSA for 1 hour and then incubated with antibodies to APAF1 (Abcam, #32372), CCNE2 (Abcam, #40890), and CASP3 (Abcam, #32351) overnight at 4 °C. The membranes were subsequently washed and then incubated for 1 hour with an anti-rabbit IgG horseradish peroxidase-linked secondary antibody (Cell signaling, #7074). The membranes were then washed and briefly incubated using with an Amersham ECL Western Blotting detection reagent (GE Healthcare, #RPN2209).

### Statistical analysis

The sample and control groups were compared using a two-tailed Student t-test. All data presented includes the standard deviation (*SD*). *P*-values of less than 0.05 were considered to be statistically significant.

## Results

### The effect of trastuzumab on cell viability

**Figure 1:**
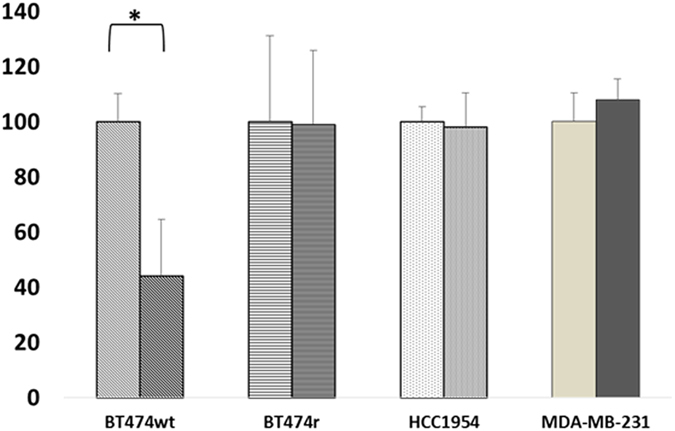
Cell viability after trastuzumab treatment. BT474 wt, BT474r, HCC1954, and MDA-MB-231 cells were exposed to 15 μg/ml of trastuzumab for 7 days and their viability was assessed using MTT assays. The percentage of viable cells was calculated with respect non-treated cells. *p < 0.05.

First we examined the effect of trastuzumab on cell viability in our HER2-positive (BT474 wt, BT474r, and HCC1954) and HER2-negative (MDA-MB-231) breast cancer cell lines. After seven days of trastuzumab treatment we observed a 40% reduction in BT474 wt proliferation (*p* = 0.01) but no changes in viability in the BT474r, HCC1954, or MDA-MB-231 cells (Fig. 1). Annexin-V assays showed that BT474 wt cell viability was reduced from 97.7% to 22.6% after trastuzumab treatment while BT474r cells were not affected by the same treatment.

### Both miR-30b and miR-26a are trastuzumab-responsive miRNAs

**Figure 2:**
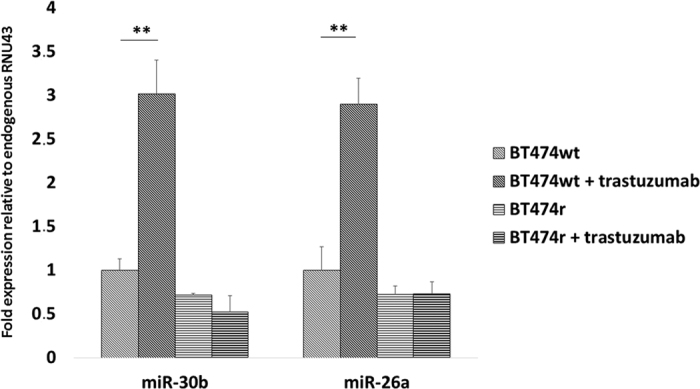
Expression levels of miR-30b and miR-26a. The levels of miR-30b and miR-26a were measured with and without trastuzumab treatment in BT474 wt (trastuzumab-sensitive) and BT474r (trastuzumab-resistant) cells. Fold-expression was calculated in relation to endogenous RNU43. **p < 0.01.

Similar to previous studies, the three HER2-positive and one HER2-negative breast cancer cell lines were exposed to trastuzumab^9^ and then miR-30b and miR-26a expression were analyzed in these cells by real-time qPCR analysis. As shown in Fig. 2, the fold-expression of miR-30b and miR-26a (relative to endogenous *RNU43*) increased after trastuzumab treatment in BT474 wt cells (~3 fold, *p* = 0.002 and *p* = 0.003 for miR-30b and miR-26a, respectively), while no significant changes in expression were observed in the other cell lines, which were all resistant to trastuzumab treatment.

### MiR-30b and miR-26a overexpression sensitize cells to trastuzumab treatment

**Figure 3:**
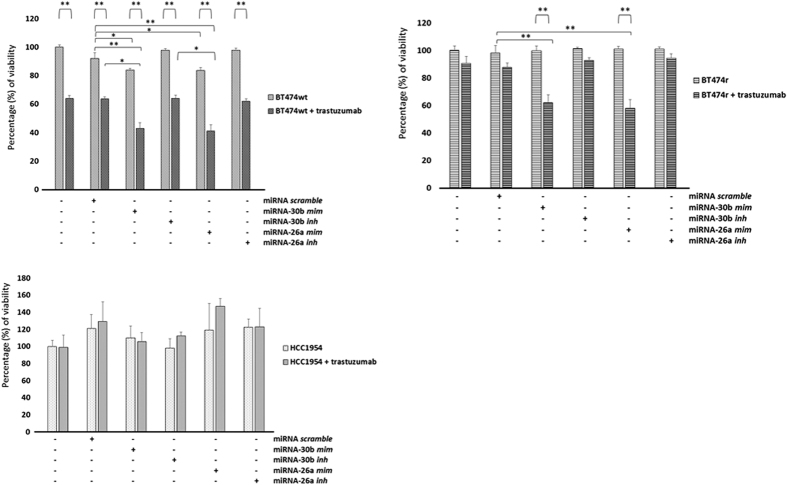
The effect of miR-30b and miR-26a overexpression or downregulation on cell viability. BT474 wt, BT474r, and HCC1954 cell lines were transfected with miR-30b and miR-26a mimetics or inhibitors and the viability of the cells was assayed after 7 days after trastuzumab treatment and under control conditions. The experiments were performed in triplicate and the values are calculated in relation to the negative control transfections with scramble miRNAs. *p < 0.05, **p < 0.01.

Transfection with miR-30b or miR-26a mimics resulted in a decrease in BT474 wt cell viability (~20% reduction, *p* = 0.036 and *p* = 0.022 for miR-30b and miR-26a, respectively) in normal growth conditions, but no change in the other cell lines. However, when trastuzumab was added alongside these miRNA mimics, viability considerably reduced in both BT474 wt (~60% reduction, *p* = 0.0012 and *p* = 0.004 for miR-30b and miR-26a, respectively) and BT474r (~40% reduction, *p* = 0.01 and *p* = 0.009 for miR-30b and miR-26a, respectively) cells (Fig. 3). No changes were observed in HCC1954 and MDA-MB-231 (data not shown). In contrast, transfection with miR-30b or miR-26a inhibitors did not affect the viability of any of the cell lines either in normal conditions or with trastuzumab treatment.

The flow cytometry viability assays showed that the percentage of viable BT474 wt cells reduced from 97.7% to 4.11% when miR-30b mimic was transfected and to 2.66% when this mimic was combined with trastuzumab treatment. MiR-30b overexpression in BT474r reduced cell viability from 97.6% to 44.8%, however this result did not differ between cohorts transfected with miR-30b mimic alone or combined with trastuzumab.

### The effect of trastuzumab on the cell cycle

**Figure 4:**
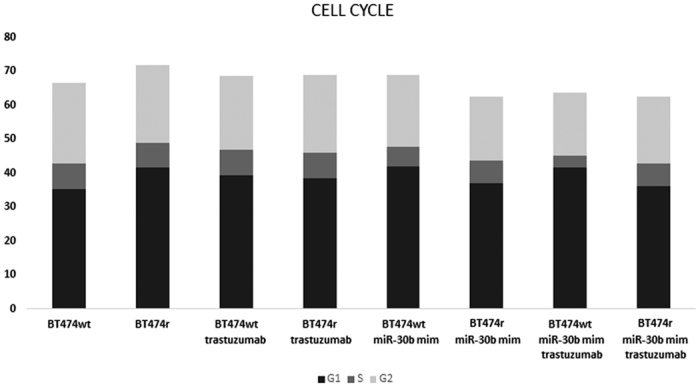
Distribution of the cell cycle phases after trastuzumab treatment. The percentage of cells in G1, S, and G2 phases was evaluated in BT474 wt and BT474r cells, with or without miR-30b overexpression, and with or without trastuzumab treatment.

Analysis of the cell cycle phases by flow cytometry (Fig. 4) showed that trastuzumab induces G1 arrest (4.7% increase compared to control) in BT474 wt cells. Transfection with miR-30b mimic induced stronger G1 arrest in BT474 wt cells (6.8% increase), which also increased when miR-30b overexpression was combined with trastuzumab treatment (6.7% increase). Proliferative (S + G2) BT474 wt cells decreased from 31.3% to 29% when trastuzumab was added further decreased to 26.87% when miR-30b mimic was also transfected, and reduced to 22.08% when combined with trastuzumab. Neither trastuzumab treatment nor miR-30b mimic transfection caused G1 cell cycle arrest in BT474r cells and BT474r proliferative cells remained the same following trastuzumab treatment, but the addition of miR-30b mimic decreased the number of BT474r proliferative (S + G2) cells by 5.13% both with and without the presence of trastuzumab.

### The impact of miR-30b and miR-26a on cell cycle genes

**Figure 5:**
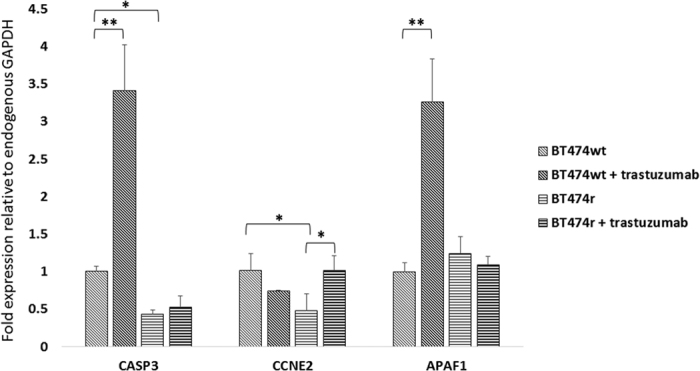
The effect of trastuzumab on *CASP3, CCNE2*, and *APAF1* expression. The levels of *CASP3, CCNE2*, and *APAF1* genes with or without trastuzumab treatment were analyzed in BT474 wt and BT474r cells. The experiments were performed in triplicate and the values were related to endogenous *GAPDH* expression. *p < 0.05, **p < 0.01.

Because both miR-30b and miR-26a seemed to be implicated in regulating cell viability, we checked how these miRNAs modulate the cell cycle and cell proliferation. First we measured the expression of the cell cycle regulator gene *CCNE2* and the two pro-apoptotic genes *APAF1* and *CASP3*, in normal conditions and after trastuzumab treatment. *CASP3* and *APAF1* showed increased expression after this treatment in BT474 wt cells (~3 fold, *p* = 0.0012 and *p* = 0.0017, respectively) but no significant differences were observed in *CCNE2* expression. In contrast, only *CCNE2* expression significantly increased (*p* = 0.032) after trastuzumab treatment in BT474r cells. Basal expression of *CASP3* and *CCNE2* was lower in resistant BT474r cells compared to wild-type BT474 wt cells (*p* = 0.018 and *p* = 0.029, respectively), whereas basal *APAF1* expression was similar in both cell lines (Fig. 5). HCC1954 cells did not show changes in *APAF, CASP3* or *CCNE2* expression (data not shown).

**Figure 6:**
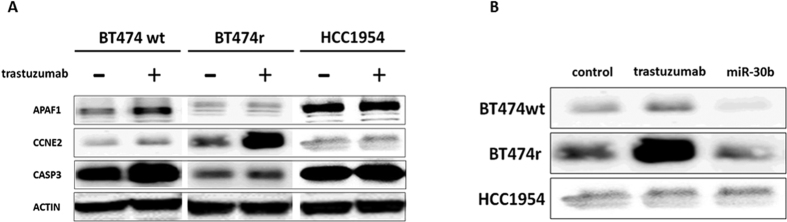
The effect of trastuzumab on CASP3, CCNE2, and APAF1 protein expression by western blot analysis. (**A**) Levels of APAF1, CCNE2, and CASP3 proteins were measured in BT474 wt, BT474r, and HCC1954 cells exposed to trastuzumab (15 μg/ml for 7 days). Actin was used as loading control. (**B**) Levels of CCNE2 protein in BT474 wt, BT474r, and HCC1954 cells transfected with miR-30b mimetic.

Western blot analysis showed that APAF1 and CASP3 proteins were highly expressed in BT474 wt cells after trastuzumab treatment, whereas CCNE2 expression did not change. In contrast, the opposite was seen in BT474r cells: APAF1 and CASP3 did not change but CCNE2 was strongly expressed. There were no changes in protein expression after treatment in the HCC1954 cell line (Fig. 6A).

**Figure 7:**
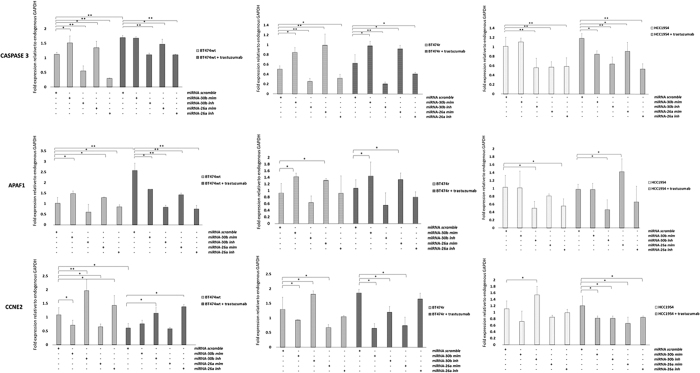
Regulation of *CASP3, CCNE2*, and *APAF*1 expression by miR-30b and miR-26a. The BT474 wt, BT474r, and HCC1954 cell lines were transfected with miR-30b and miR-26a mimetics or inhibitors and the expression levels of *CASP3, CCNE2*, and *APAF1* were evaluated by quantitative PCR after trastuzumab treatment or under control conditions. The experiments were performed in triplicate and the values were normalized to endogenous *GAPDH* gene expression. *p < 0.05, **p < 0.01.

We also checked gene expression levels in these cells lines after miR-30b or miR-26a mimic or inhibitor transfection (Fig. 7). In normal conditions, transfection with either miR-30b or miR-26a mimic resulted in a significant increase in the expression of the pro-apoptotic *CASP3* and *APAF1* genes in BT474 wt (*CASP3: p* = 0.026 and *p* = 0.032, *APAF1: p* = 0.035 and *p* = 0.042, for miR30b and miR-26a respectively) and BT474r cells (*CASP3: p* = 0.034 and *p* = 0.019, *APAF1: p* = 0.041 and *p* = 0.034, for miR30b and miR-26a respectively), but not in HCC1954 cells. In addition, *CCNE2* expression was also significantly downregulated in BT474 wt and BT474r cell lines after miR-30b or miR-26a mimic transfection (BT474 wt: *p* = 0.042 and *p* = 0.035, BT474r: *p* = 0.032 and *p* = 0.041, for miR30b and miR-26a respectively), but not in HCC1954. In contrast, these results were reversed when the cells were transfected with miR-30b or miR-26a inhibitors: the *CASP3* and *APAF1* genes were downregulated while *CCNE2* was upregulated (BT474 wt: *p* = 0.002 and *p* = 0.021, for miR-30b and miR-26a respectively; BT474r: p = 0.034 for miR-30b and not significant changes for miR-26a). In the same way, transfection with miR-30b mimic produced a decrease in CCNE2 protein expression in both BT474 wt and BT474r cell lines compared to controls (Fig. 6B).

When trastuzumab was combined with the miR-30b or miR-26a mimics in BT474 wt and BT474r cells, *CASP3* and *APAF1* expression significantly increased and *CCNE2* expression significantly decreased in comparison to control cells reaching similar levels of the mimics without trastuzumab (Fig. 7). However, this expression pattern appeared to be attenuated both in sensitive (BT474 wt) and resistant (BT474r) cell lines when trastuzumab treatment was combined with miR-30b or miR-26a inhibitors.

### *CCNE2* is involved in cell viability

**Figure 8:**
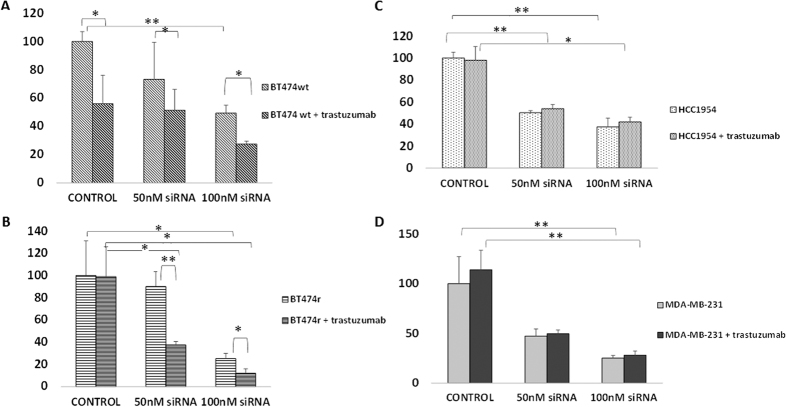
*CCNE2* silencing sensitizes BT474r cells to trastuzumab treatment. *CCNE2* gene was silenced using siRNA in BT474 wt (**A**), BT474r (**B**), HCC1954 (**C**), and negative control MDA-MB-231 (**D**), and cell viability was assessed by MTT with and without trastuzumab treatment (15 μg/ml for 7 days) *p < 0.05, **p < 0.01.

*CCNE2* silencing by siRNA (100 nM) transfection led to a decrease in cell proliferation in all of the breast cancer cell lines to ~40% viability (*p* = 0.001) in B474 wt, ~25% viability (*p* = 0.05) in BT474r, ~40% viability (*p* = 0.0005) in HCC1954, and ~20% viability (*p* = 0.003) in MDA-MB-231 cells compared to their respective controls (Fig. 8).

### *CCNE2* expression is involved in acquired trastuzumab resistance

We compared cell viability in trastuzumab-treated and non-treated cell lines transfected with siRNA using MTT assays. As shown in Fig. 8, BT474 wt cells were initially sensitive to trastuzumab (~50% viability vs. control, *p* = 0.05) even when also transfected with 50 nM siRNA (~50% viability), but cell viability reduced to 25% when 100 nM siRNA was transfected in combination with trastuzumab (*p* = 0.02 vs. non-treated sample). BT474r was initially resistant to trastuzumab (~100% viability) but viability reduced to ~25% (*p* = 0.03) and ~10%, (*p* = 0.01) respectively, after transfection with 50 nM or 100 nM siRNA, respectively in combination with trastuzumab, showing an overall 50% decrease in viability (*p* = 0.03 vs. non-treated samples). Thus, according to the criteria set out in the Materials and Methods, this cell line was considered to be trastuzumab-sensitive after transfection with 50 nM or 100 nM *CCNE2* siRNA. HCC1954 and MDA-MB-231 cells were resistant to trastuzumab and remained so after *CCNE2* silencing (~40% and ~25% final viability, respectively), representing a non-significant change compared to non-treated samples in both cases.

## Discussion

HER2+ breast tumor treatment has been greatly improved by anti-HER2 agents like trastuzumab; however, primary and secondary resistance is a substantial problem^13^. To investigate the possible mechanisms behind this resistance we must strive to understand the pathways and mechanisms modified by trastuzumab treatment. Altered miRNA profiles have been identified in several cancers in relation to malignancy and prognosis, and in metastatic breast cancer tumors miR-21 and miR-10b have been shown to be upregulated^14^. Some miRNAs, such as miR-34a or miR-221, are related to treatment response and are involved in docetaxel and tamoxifen response^15^,^16^, while miR-30b and miR-26a are upregulated in HER2+ BT474 wt cells when treated with trastuzumab^9^. Following this line of investigation, we demonstrated that this upregulation occurs in BT474 wt cells but not in acquired-resistance BT474r cells generated by our group or in the innately-resistant HCC1954 cell line. Thus, upregulation of these miRNAs may be a marker for sensitivity to trastuzumab treatment.

In addition to validating the miR-26a and miR-30b-mediated reduction in cell viability in BT474 wt cells first shown by Ichikawa *et al*., we also investigated the effect of miR-26a and miR-30b mimics and inhibitors, either alone or combined with trastuzumab, in cell lines that are either sensitive or resistant to this drug. Our results showed that while miR-26a and miR-30b mimics alone only had an effect in BT474 wt cells, when combined with trastuzumab they decreased cell proliferation in both the BT474 wt and BT474r cell lines, thus suggesting that these miRNAs are related to the acquisition of trastuzumab resistance. In addition, cell viability and cell cycle analyses confirmed previous results which showed that trastuzumab^17^ and miR-30b mimics^9^ decrease BT474 wt cell viability and produce G1 arrest, while trastuzumab does not affect the cell cycle in BT474r cells^10^. We also revealed that miR-30b mimics, alone or in combination with trastuzumab, decrease the number of proliferative BT474r cells.

Given the aforementioned effects of miR-30b and miR-26a (which are upregulated in response to trastuzumab) we tried to identify some of their cell cycle or apoptosis-related target genes. Previous studies linked the response to EGFR tyrosine kinase treatment to pro-apoptotic genes such as caspases and *APAF1* in breast^18^ and other cancers^19^. Thus, we studied the effect of trastuzumab on pro-apoptotic genes and showed that *CASP3* and *APAF1* were upregulated at the gene and protein level in BT474 wt cells when treated with trastuzumab, but that the same effect was not observed in trastuzumab-resistant cell lines. This finding suggests that the apoptotic processes induced by trastuzumab may be mediated in part by the APAF1 apoptosis pathway and that cell lines which do not overexpress this gene after trastuzumab treatment have higher proliferation and survival rates^19^.

**Figure 9:**
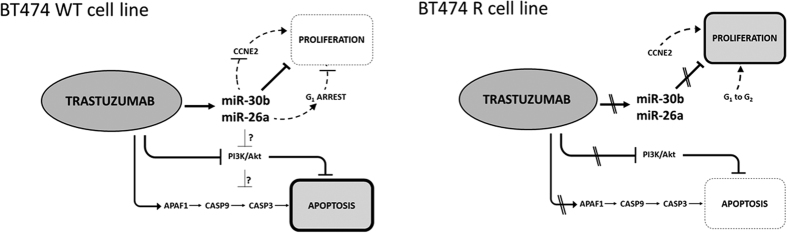
Schematic representation of trastuzumab response mechanism proposed for BT474 wt (sensitive) and BT474r (resistant) cell lines.

APAF1, activated via CARD (caspase recruitment domain), initiates the activation of caspases such as caspase-3^20^,^21^. Given that trastuzumab induces miR-30b and miR-26a upregulation, as well as CASP3 and APAF1 upregulation in BT474 wt cells, we tried to identify any relationships between these miRNAs and genes. Transfection experiments demonstrated that upregulation of miR-30b and miR-26a with mimics induced *CASP3* and *APAF1* overexpression in BT474 wt and BT474r cells, while inhibitors of these miRNAs decreased *CASP3* and *APAF1* expression independently of the presence of trastuzumab. Thus, these results suggest that apoptotic *CASP3* and *APAF1* genes may be indirectly regulated by miR-30b and miR-26a through a previously undescribed intermediate mechanism (Fig. 9).

It is known that PI3K/Akt activation is essential for HER2-mediated apoptosis suppression^22^,^23^; activation of the PI3K/Akt pathway by different mechanisms has been described both in HER2+ breast cancer patients with trastuzumab resistance^24^ and in BT474r cells^12^. Indeed, trastuzumab treatment inhibits the PI3K/Akt pathway by Akt dephosphorylation^25^. Given that activated Akt pathways inhibit *APAF1* expression and its binding with *CASP9* (which in turn regulates *CASP3* expression), it seems logical that inactivation of the Akt pathway by trastuzumab in BT474 wt cells results in increased *APAF1, CASP3*, and *CASP9* expression which does not occur in resistant cell lines with active Akt^26^. Indeed, we confirmed that *CASP9* is also significantly expressed in BT474 wt cells, but not in BT474r cells, after trastuzumab treatment (Supplementary Figure 1).

Previous studies have proposed *CCNE2* as a miR-30b target gene^9^, and have shown the role of CCNE2 in trastuzumab-acquired resistance^10^. Here, we not only demonstrated that *CCNE2* is a miR-30b target gene (because both the gene and its protein were underexpressed when BT474 wt and BT474r cells were transfected with miR-30b mimic and increased when they were transfected with its inhibitor), but also that *CCNE2* is a miR-26a target. In addition, *CCNE2* expression is related to trastuzumab response^10^: while *CCNE2* expression reduced in BT474 wt cell lines treated with trastuzumab, it increased in BT474r cells, indicating that *CCNE2* overexpression could be a mechanism of trastuzumab resistance. However, given that the HCC1954 cell line with innate trastuzumab resistance did not overexpress *CCNE2*, we propose that it is a mechanism of acquired-resistance only.

Our findings agree with those by Scaltriti and colleagues (2012), who transfected CCNE2 siRNA into the trastuzumab-resistant BT474 cell line generated in-house, and showed that resistance to this drug depends on CCNE2 overexpression in these cells. We confirmed these results in our resistant cell line, BT474r, and also demonstrated that once CCNE2 was silenced, the viability of these cells reduced by more than 20% which, according to O’Brien *et al*., should be considered drug-sensitive^12^. Although CCNE2 could be part responsible for acquired resistance in BT474r cells, it seems that it is not related to the innate resistance displayed by HCC1954 cell lines because transfection with CCNE2 siRNA decreased proliferation but did not affect trastuzumab response.

Given the relevance of the Akt pathway and *CASP3* and *APAF*1 genes in trastuzumab response, and the implication of miR-26a and miR-30b in treatment sensitivity, it is possible that these apoptotic genes are trans-regulated by a direct miR-26a or miR-30b target gene. If activated Akt could be used as a trastuzumab-treatment response marker in HER2+ breast cancer^26^,^27^,^28^, *CASP3* and *APAF1* overexpression may also represent possible treatment-sensitivity markers. Thus, it would be interesting to go deeper into the relationship between the PI3K/AKT pathway and the miRNAs and genes relevant in this context in order to validate this proposed mechanism. Similarly, evaluating genes and proteins which are altered in samples from trastuzumab-resistant breast cancer patients would also provide valuable information. Despite these limitations, we propose that *CCNE2* overexpression may represent a potential biomarker of acquired trastuzumab resistance in patients with HER2+ breast cancer. Finally, new therapies combining trastuzumab with CCNE2 inhibitors might represent a new strategy for overcoming acquired resistance to this drug.

## Additional Information

**How to cite this article:** Tormo, E. *et al*. The role of miR-26a and miR-30b in HER2+ breast cancer trastuzumab resistance and regulation of the CCNE2 gene. *Sci. Rep.* **7**, 41309; doi: 10.1038/srep41309 (2017).

**Publisher's note:** Springer Nature remains neutral with regard to jurisdictional claims in published maps and institutional affiliations.

## Supplementary Material

Supplementary Figure 1
