# Supplementary material for: The role of miR-26a and miR-30b in HER2+ breast cancer trastuzumab resistance and regulation of the CCNE2 gene
Source: Sci Rep. 2017 Jan 25;7:41309. doi: 10.1038/srep41309 (PMC5264595; doi:10.1038/srep41309)
Supplement: Supplementary Figure 1 [file srep41309-s1.pdf]

Title: The role of miR-26a and miR-30b in HER2+ breast cancer trastuzumab resistance and regulation of the *CCNE2* gene

Authors: Eduardo Tormo, Anna Adam-Artigues, Sandra Ballester, Begoña Pineda, Sandra Zazo, Paula González-Alonso, Joan Albanell, Ana Rovira, Federico Rojo, Ana Lluch and Pilar Eroles

Supplementary figure 1

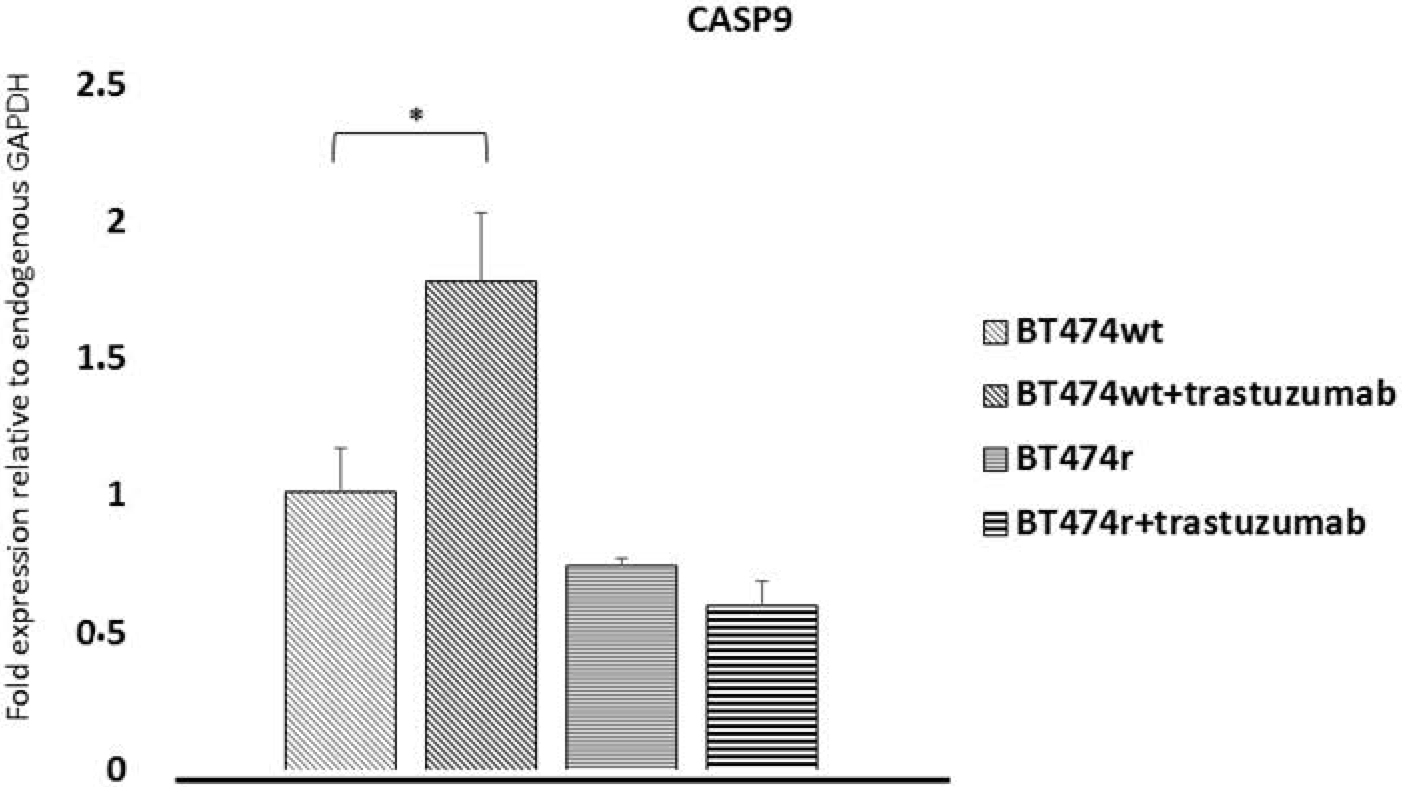

Effect of trastuzumab in *CASP9* gene expression

The levels of *CASP9* with or without trastuzumab treatment were analyzed in BT474wt and BT474r cell lines. The experiments were performed in triplicate and the values related to endogenous *GAPDH* expression. * p = 0.013
